# Histamine Is an Inducer of the Heat Shock Response in SOD1-G93A Models of ALS

**DOI:** 10.3390/ijms20153793

**Published:** 2019-08-03

**Authors:** Savina Apolloni, Francesca Caputi, Annabella Pignataro, Susanna Amadio, Paola Fabbrizio, Martine Ammassari-Teule, Cinzia Volonté

**Affiliations:** 1 IRCCS Santa Lucia Foundation, 00143 Rome, Italy; 2 IRCCS Istituto di Ricerche Farmacologiche Mario Negri, 20156 Milan, Italy; 3 National Research Council, Institute for Systems Analysis and Computer Science, 00185 Rome, Italy

**Keywords:** dendritic spines, heat shock proteins, histamine, microglia, motor neurons, SOD1-G93A

## Abstract

(1) Background: Amyotrophic lateral sclerosis (ALS) is a multifactorial non-cell autonomous disease where activation of microglia and astrocytes largely contributes to motor neurons death. Heat shock proteins have been demonstrated to promote neuronal survival and exert a strong anti-inflammatory action in glia. Having previously shown that the pharmacological increase of the histamine content in the central nervous system (CNS) of SOD1-G93A mice decreases neuroinflammation, reduces motor neuron death, and increases mice life span, here we examined whether this effect could be mediated by an enhancement of the heat shock response. (2) Methods: Heat shock protein expression was analyzed in vitro and in vivo. Histamine was provided to primary microglia and NSC-34 motor neurons expressing the SOD1-G93A mutation. The brain permeable histamine precursor histidine was chronically administered to symptomatic SOD1-G93A mice. Spine density was measured by Golgi-staining in motor cortex of histidine-treated SOD1-G93A mice. (3) Results: We demonstrate that histamine activates the heat shock response in cultured SOD1-G93A microglia and motor neurons. In SOD1-G93A mice, histidine augments the protein content of GRP78 and Hsp70 in spinal cord and cortex, where the treatment also rescues type I motor neuron dendritic spine loss. (4) Conclusion: Besides the established histaminergic neuroprotective and anti-inflammatory effects, the induction of the heat shock response in the SOD1-G93A model by histamine confirms the importance of this pathway in the search for successful therapeutic solutions to treat ALS.

## 1. Introduction

Amyotrophic lateral sclerosis (ALS) is one of the most common neuromuscular diseases (2–3 cases per 100,000 individuals/year), characterized by the selective loss of upper and lower motor neurons in the cortex, brain stem and ventral horns of the spinal cord. Currently, the glutamate release inhibitor riluzole, and the antioxidant edaravone, are the only two FDA-approved drugs for ALS, although they are restricted to symptomatic therapy for relieving spasticity and slightly prolonging life expectancy [1]. The majority of ALS cases are sporadic with an unknown etiology, but about 20% of familial forms are associated with a large number of mutations in superoxide dismutase 1 (*SOD1*) gene [2]. ALS has a marked multifactorial as well as non-cell autonomous nature, where several mechanisms contribute to the pathogenesis, among which is the activation of glia that largely influences the death of motor neurons [3,4].

Heat shock proteins (Hsps) have been shown to be powerful inhibitors of apoptosis by directly interacting with several members of the apoptotic pathway, and by exerting an anti-inflammatory action in glia [5], thereby culminating in neuronal survival [6]. In ALS, the motor neurons have an intrinsic deficit in the ability to activate the heat shock response (HSR) and do not readily up-regulate the expression of Hsps, such as Hsp70, in response to stress, resulting in protein aggregates formation, axonal transport deficit and ultimately cell death [7]. Indeed, many strategies aimed at increasing intracellular Hsp70 levels have been successfully tested in a variety of neurodegeneration models including ALS, and notably the HSR-inducer arimoclomol is presently in clinical trials for ALS patients [8]. Furthermore, it has been proposed that mutations in the SOD1 protein induce the disease by a toxic gain of function that includes the dysregulation of the HSR-apoptosis axis. As a result, the overexpression of anti-apoptotic proteins such as Bcl-2 and Bcl-xL attenuates neurodegeneration produced by the SOD1-G93A mutation [9], thus highlighting the Bcl family as crucial targets of ALS pathogenesis.

Enhanced Hsps expression can lead to effective clearance of aggregated proteins by activating autophagy [10], a highly conserved mechanism by which eukaryotic cells degrade intracellular damaged components and protein aggregates. Not surprisingly, abnormalities in autophagy have been implicated in chronic neurodegenerative conditions comprising ALS, where both patients and animal models are characterized by defects in the expression of autophagy-related proteins such as Beclin-1 and sequestosome-1/p62 (SQSTM1/p62) [11].

Histamine is a biogenic amine synthesized by decarboxylation of L-histidine by histidine decarboxylase and catabolized by histamine N-methyltransferase or by diamine oxidase, enzymes that are distributed essentially in all central nervous system (CNS) areas. In the CNS, histamine regulates the sleep-wake cycle, nociception, motor circuits and neuroimmune functions [12]. As proven by human post-mortem and animal model studies, the histaminergic system becomes altered in the CNS when neuroinflammation exacerbates the outcome for instance of traumatic brain/spinal cord injury, multiple sclerosis, Alzheimer’s, Parkinson’s and Huntington’s diseases, and several histamine-related drugs are in clinical trial for these pathologies [13,14,15].

Our previous studies have shown that histamine-related genes are dysregulated in the spinal cord and cortex of both sporadic ALS patients and SOD1-G93A mice during different phases of disease progression. Furthermore, a pharmacological approach increasing the CNS histamine content in SOD1-G93A mice ameliorates various pathological features of ALS, most importantly life span, motor performance, neuroinflammation, muscle atrophy and motor neuron death [16]. Finally, histamine induces an anti-inflammatory profile in SOD1-G93A microglia, and sustains the survival of SOD1-G93A motor neurons in culture [16,17].

HSR, and particularly Hsp70, plays an important role in the cellular processes implicated in ALS including stress granule formation, autophagy, proteasomal degradation and inflammatory signaling [18]. As an attractive therapeutic strategy for diseases, such as ALS, consists of boosting the Hsp levels in neurons and glial cells [10,19], in the present work we investigate if histamine signaling exerts its neuroprotective action through the HSR triggered in vitro in motor neurons and microglia cultures, and in vivo in spinal cord and cortex from symptomatic SOD1-G93A mice.

## 2. Results

### 2.1. Histamine Modulates the Hsps Response in SOD1-G93A Primary Microglia

**Figure 1:**
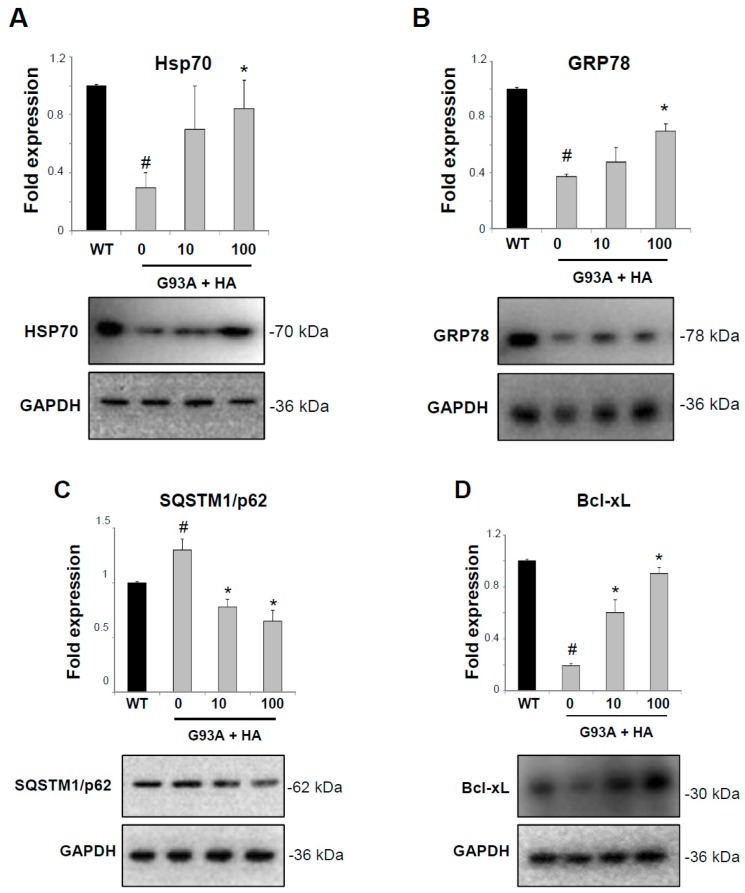
Histamine induces the heat shock response in SOD1-G93A microglia. Representative western blots and quantification of Hsp70 (**A**), GRP78 (**B**), SQSTM1/p62 (**C**) and Bcl-xL (**D**) in wild-type (WT) and SOD1-G93A microglia treated with histamine (10–100 μM) for 6 h. GAPDH was used for protein normalization. HA = histamine. Data represent mean ± S.E.M. (*n* = 3 independent experiments). Statistical significance was calculated by ANOVA, as referred to WT cells, # *p* < 0.05 or to SOD1-G93A untreated cells, * *p* < 0.05.

Hsps are important regulators of inflammatory conditions, by acting in microglia and astrocytes to inhibit nuclear factor kappa-light-chain-enhancer of activated B cells (NF-κB), and to prevent the over-production of inflammatory mediators such as nitric oxide (NO) synthase and cycloxygenase-2 [6]. We have previously demonstrated that histamine is able to polarize the SOD1-G93A microglia towards an anti-inflammatory phenotype both in vitro and in vivo, by decreasing NF-κB and detrimental M1-like markers, while increasing beneficial M2-like markers [16,17]. Here, we have analyzed the effects of histamine on Hsps proteins. SOD1-G93A microglia show a robust decrease in Hsp70 and GRP78 expression compared to wild-type (WT) microglia (Figure 1A,B), confirming that SOD1-G93A mutation impairs the HSR cellular defense mechanism. However, the histamine treatment at 10–100 μM (concentrations that are proved not to be cytotoxic in our system, data not shown) in SOD1-G93A microglia significantly restores this impairment by augmenting both Hsp70 and GRP78 protein levels (Figure 1A,B). In addition, a slight but statistically significant increase in SQSTM1/p62 autophagic protein is observed in SOD1-G93A compared to WT microglia (Figure 1C), confirming that SOD1-G93A mutation interferes with this degradation machinery [20]. Of note, HA treatment in ALS microglia significantly diminishes SQSTM1/p62 levels (Figure 1C). As an enhanced expression of Hsps also inhibits apoptosis [21], we next analyzed the level of the anti-apoptotic Bcl-xL protein. As shown in Figure 1D, SOD1-G93A microglia show a strong reduction in the anti-apoptotic Bcl-xL with respect to WT microglia, but this effect is in part dose-dependently abolished by histamine at 10–100 μM.

### 2.2. Histamine Modulates the Hsps Response in NSC-G93A Motor Neuron Cells

**Figure 2:**
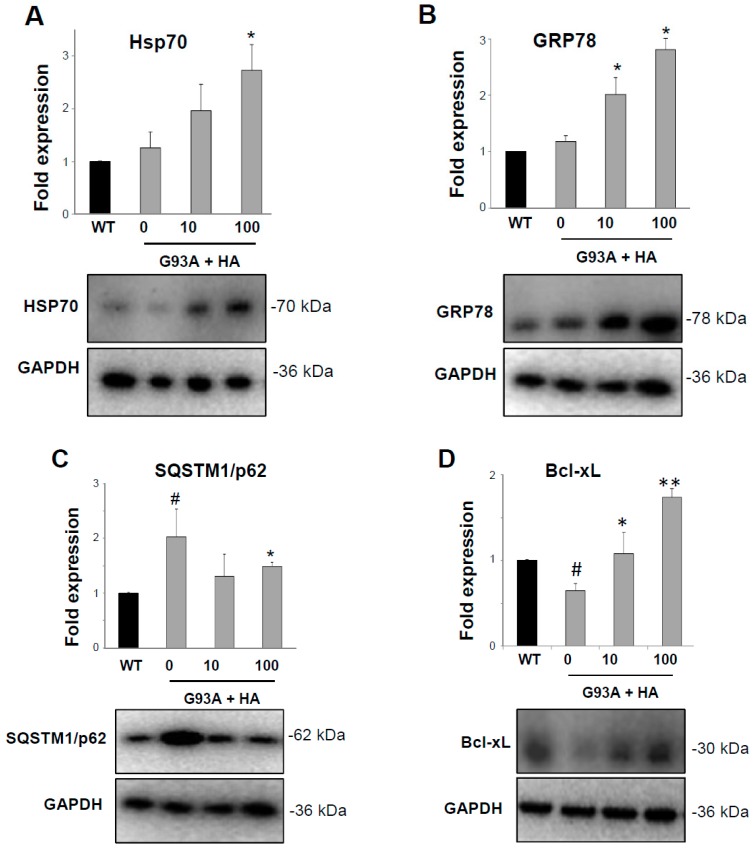

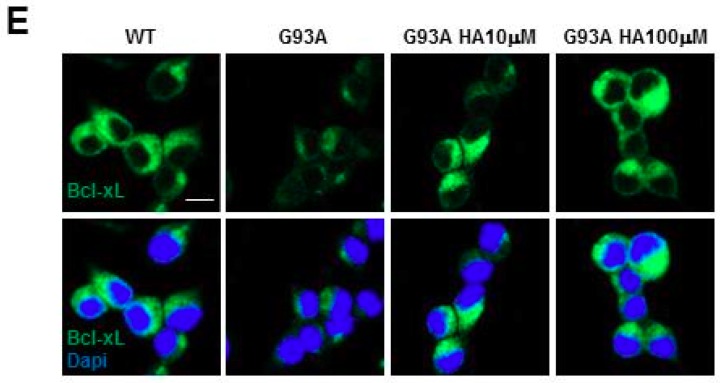
Histamine induces the Heat Shock Response in NSC-G93A motor neuron cells. Representative western blots and quantification of Hsp70 (**A**), GRP78 (**B**), SQSTM1/p62 (**C**) and Bcl-xL (**D**) in differentiated NSC-WT and NSC-G93A cells treated with histamine (10–100 μM) for 48 h. GAPDH was used for protein normalization. HA = histamine. Data represent mean ± S.E.M. (*n* = 3 independent experiments). Statistical significance was calculated by ANOVA, as referred to NSC-WT cells, # *p* < 0.05 or to NSC-G93A untreated cells, * *p* < 0.05; ** *p* < 0.01. (**E**) Representative immunofluorescence images of Bcl-xL (green) in NSC-WT and NSC-G93A cells treated with histamine (10–100 μM). Dapi for nuclei staining is shown in blue. Scale bar: 20 μm.

We have previously shown that histamine elicits neuroprotective actions on SOD1-G93A motor neurons, particularly by restoring a proper mitochondrial metabolism and activating the AKT/ERK1/2 pro-survival pathway [16]. As an increased content of Hsps is a well-known neuroprotective mechanism in ALS [6], we investigated whether histamine directly affects Hsps proteins also in motor neuron-like cells expressing the SOD1-G93A mutation. Primary motor neurons are not a pure and homogeneous cell culture; thus, we have adopted differentiated NSC-34 cells that constitute a well-characterized model system for studying ALS-related mechanisms, and in particular damage induced by mutant SOD1 [22,23]. As shown in Figure 2A,B, histamine treatment at 10–100 μM (concentrations that are proved not to be cytotoxic in our system, data not shown) stimulates the expression of both Hsp70 and GRP78 proteins in differentiated NSC-G93A cells under serum starvation, an in vitro condition that closely resembles the growth factors deprivation occurring in motor neurons during ALS. Moreover, the presence of the SOD1-G93A mutation causes a significant accumulation of the SQSTM1/p62 autophagic protein with respect to WT cells (Figure 2C), and importantly, this effect is prevented by histamine treatment (Figure 2C). In accordance with previous studies [23], Bcl-xL protein is strongly reduced in NSC-G93A with respect to WT cells (Figure 2D,E), but histamine (10–100 μM) restores high levels of Bcl-xL, as demonstrated by both western blot (Figure 2D) and immunofluorescence analysis, showing an increased Bcl-xL staining mainly in the cytosolic compartment (Figure 2E).

### 2.3. Histaminergic Signaling Activates the Hsps Response in Spinal Cord from SOD1-G93A Symptomatic Mice

Previous studies have shown that animal models of ALS lack the stress-induced up-regulation of Hsps [7] and that a late stage treatment with drugs activating the HSR delays disease progression and prevents protein aggregation in the SOD1 mouse model of ALS [24]. We have previously demonstrated that the CNS permeable histamine precursor histidine increases motor neuron survival and decreases neuroinflammation in the spinal cord, when administered to SOD1-G93A mice from the onset of the disease symptoms until end stage [16]. In order to investigate whether the beneficial histaminergic signaling also proceeds in vivo through the Hsps axis, we evaluated the levels of Hsp70 and GRP78 in the spinal cords of SOD1-G93A mice after the treatment with 100 mg/kg histidine. At this dose, histidine treatment shows no evidence of distress and toxic effects in mice, as evaluated by daily inspection of body appearance and measurement of body weight. Moreover, histidine-treated mice present no differences on postmortem gross examination compared with saline-treated mice.

**Figure 3:**
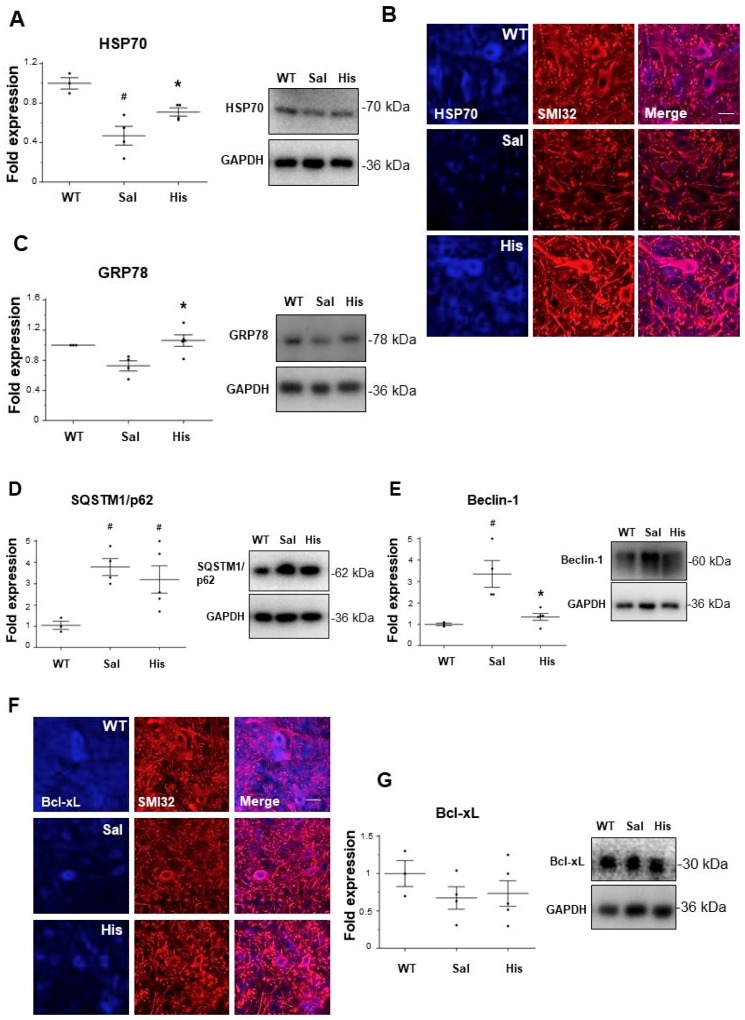
Histidine modulates the expression of Hsp70, GRP78, Beclin-1 and Bcl-xL in the spinal cord of symptomatic SOD1-G93A mice. Representative western blots and quantification of Hsp70 (**A**), GRP78 (**C**), SQSTM1/p62 (**D**), Beclin-1 (**E**) and Bcl-xL (**G**) in saline (*n* = 4) and histidine-treated 100 mg/kg (*n* = 5) SOD1-G93A mice. GAPDH was used as a loading control. Data represent mean ± S.E.M. Statistical significance was calculated by ANOVA, as referred to WT, # *p* < 0.05 or to SOD1-G93A saline-treated mice, * *p* < 0.05. (**B**) Representative immunofluorescence images of Hsp70 (blue) and SMI32 (red) in saline (*n* = 4) and 100 mg/kg histidine-treated (*n* = 5) SOD1-G93A mice. Scale bar: 100 μm. (**F**) Representative immunofluorescence images of Bcl-xL (blue) and SMI32 (red) in saline (*n* = 4) and 100 mg/kg histidine-treated (*n* = 5) SOD1-G93A mice. Scale bar: 100 μm.

As observed at the symptomatic phase of the disease, there is a loss of Hsp70 (Figure 3A) and GRP78 (Figure 3C) content in the spinal cord homogenates of SOD1-G93A compared to WT mice. The treatment with histidine significantly increases both Hsp70 and GRP78 levels in SOD1-G93A with respect to saline-treated mice (Figure 3A,C). The increase of Hsp70 in motor neurons of histidine-treated compared to saline-treated SOD1-G93A mice is further confirmed by immunofluorescence and confocal analysis (Figure 3B). We next evaluated the levels of autophagic proteins SQSTM1/p62 and Beclin-1, known to accumulate in the spinal cord of symptomatic SOD1-G93A mice [11]. As shown in Figure 3D, SQSTM1/p62 is increased in SOD1-G93A with respect to WT mice, but there were no statistically significant differences between saline and histidine groups. However, the expression of Beclin-1 is higher in SOD1-G93A compared to WT mice, and in this case the histidine treatment significantly restores its low basal content (Figure 3E). Finally, by looking at the anti-apoptotic Bcl-xL expression, immunofluorescence analysis shows a strong decrease in Bcl-xL staining in SOD1-G93A motor neurons compared to WT, which is in part restored after the in vivo treatment with histidine (Figure 3F), although the modulation of Bcl-xL in the total spinal cord lysate is not statistically different between WT and SOD1-G93A groups, when analyzed by western blot (Figure 3G).

### 2.4. Histaminergic Signaling Activates the Hsps Response and Prevents Dendritic Spine Loss in Motor Cortex from SOD1-G93A Symptomatic Mice

**Figure 4:**
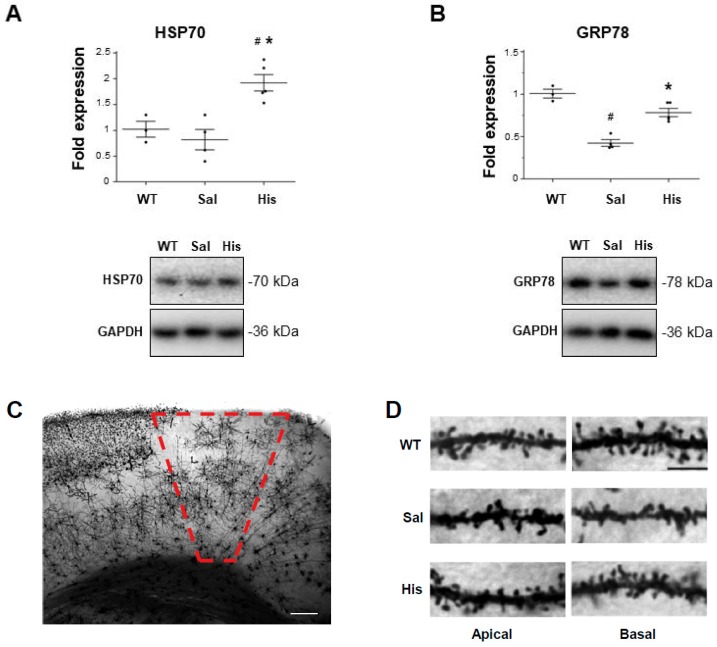

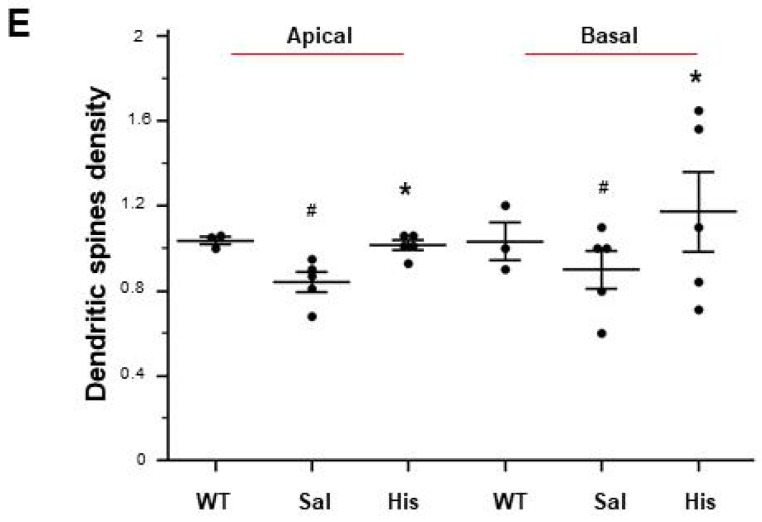
Histidine increases Hsp70 and GRP78 and dendritic spine density in the cortex of symptomatic SOD1-G93A mice. Representative western blots and quantification of Hsp70 (**A**) and GRP78 (**B**) in saline (*n* = 4) and 100 mg/kg histidine-treated (*n* = 5) SOD1-G93A mice. GAPDH was used as a loading control. Representative images of Golgi-stained motor cortex (**C**), 5× magnification; Scale bar: 50 μm) and of dendritic segments (apical and basal) of Layer V M1 pyramidal neurons from WT, saline (*n* = 4) and 100 mg/kg histidine-treated (*n* = 5) SOD1-G93A mice (**D**), 100× magnification; Scale bar: 5 μm); spine density (n spines/μm) was counted in apical and basal compartment of Layer V pyramidal neurons of M1 (**E**). Data represent mean ± S.E.M. Statistical significance was calculated by ANOVA, as referred to WT, # *p* < 0.05 or to SOD1-G93A saline-treated mice, * *p* < 0.05.

Previous data have shown that functional and structural neuronal alterations occur as a pathological feature also in the brain of SOD1-G93A mice [25,26]. Thus, we investigated whether histidine treatment also affects HSR at this level. As shown in Figure 4A, in brain homogenates from SOD1-G93A symptomatic mice, histidine treatment increases Hsp70 protein expression with respect to saline-treated mice. Moreover, SOD1-G93A mice show a strong decrease in GRP78 expression compared to WT mice and histidine in part abolishes this effect (Figure 4B). In SOD1-G93A mice, synaptic defects and dendritic spine loss start to be detected in cortical neurons concurrently with the onset of motor alterations [27,28], and then worsen in association with the full symptomatology. Previous results showing that histamine elicits neuroprotective actions on SOD1-G93A motor neurons [16], together with the present observation that histamine activates Hsps mechanisms, prompted us to examine whether histaminergic signaling could contrast dendritic spine loss in type I cortical motor neurons. Statistical comparisons of spine density in the apical and basal dendrite compartment of Golgi-stained M1 pyramidal neurons (Figure 4C) from WT, SOD1-G93A, and SOD1-G93A histidine-injected mice reveals significant group differences (Figure 4D,E; basal dendrites: F(2, 70) = 37,045, *p* < 0.05); apical dendrites: F(2, 71) = 53,876, *p* < 0.01). Post-hoc pairwise comparisons show that spine density is significantly lower in SOD1-G93A mice, with respect not only to WT mice, but also to SOD1-G93A histidine-injected mice (Figure 4D,E; *p* < 0.05 for basal dendrites; *p* < 0.01 for apical dendrites). Notably, the WT and SOD1-G93A histidine-injected groups exhibit similar spine density scores, thus indicating that histidine entirely prevents dendritic spine loss in motor cortex neurons.

## 3. Discussion

In ALS, glial cells and microglia participate in disease progression by sustaining a vicious cycle of neuroinflammation that leads to impaired motor neurons function and cell death [29]. The present work stands from previous results showing that histamine reduces the pro-inflammatory profile of SOD1-G93A microglia by decreasing NF-κB, NADPH oxidase 2, inducible NO synthase and simultaneously induces an anti-inflammatory phenotype by augmenting the expression of arginase 1, CD163, CD206, IL-6, IL10 and purinergic P2Y12 receptors [16,17]. In addition, histamine protects SOD1-G93A motor neuron-like cells from serum deprivation-induced cell death, by activating the AKT/ERK1/2 pathway and ameliorating mitochondrial metabolism with ATP production [16].

An important aspect of glial function is to respond to environmental stressors to confer protection to neurons through the intracellular HSR. Thus, in the present work, we investigated if the histaminergic challenge could increase the HSR in both neurons and glia, thus constituting an attractive therapeutic strategy for ALS [7,10]. Indeed, Hsp70 expression was reduced in the post-mortem tissues of ALS patients, while in the spinal cord Hsp70 protein accumulates and aggregates, thus leading to a deficit in cytosolic Hsps [18]. In addition to Hsp70, Hsp40 and HSF1 protein levels decreased in post-mortem tissue from patients with sporadic ALS, and in a mouse model of TDP-43-linked ALS [30]. Remarkably, depletion of cytosolic Hsps not only occurs in the diseased CNS, but also in aged tissues, thus apparently contributing to the development of neurodegenerative diseases including ALS [31]. Our results not only confirm that Hsp70 and GRP78 are involved in the pathogenic mechanisms of ALS, but also that ALS motor neurons have an impaired ability to induce the heat shock response under stress conditions [32,33]. Above all, we establish that an increased histaminergic signaling stimulates Hsp70 and GRP78 expression not only in vitro in SOD1-G93A motor neurons and microglia, but, importantly, in spinal cord and cortex from symptomatic SOD1-G93A mice, although future experiments are required to dissect the direct mechanism of the histidine in vivo effects. Moreover, because the presence of Hsp70 in the serum is suggested to be a biomarker for inflammatory processes in ALS patients [34], future experiments might consider measuring Hsp70 levels in the serum of ALS mice during histidine treatments.

As HSR activation is known to control the inflammatory pathway in glia by decreasing the levels of NF-κB and the production of NO [6], and because the anti-inflammatory action exerted by histamine in microglia indeed works through NF-κB and NO signaling [16,17], we suggest that the activation of Hsp70 and GRP78 might be strictly linked to the anti-inflammatory action exerted by histamine in microglia. Having previously demonstrated that the activation of the histaminergic system elicits overall beneficial effects on disease progression of ALS mice [16], the present results suggest that protective histamine signaling might proceed through the HSR pathway. Future studies by the use of SOD1-G93A transgenic mice depleted of either the histidine decarboxylase enzyme or the histamine H1R-H4R receptors will aim to prove this hypothesis that is in accordance with the administration of HSR-targeting drugs as therapeutics for ALS [10]. Thus, our work would add histamine to the list of compounds stimulating the production of Hsps, such as Celastrol [35], BGP-15 and arimoclomol [10], which are encouraging to counteract the progression of the disease. Of relevance, arimoclomol has not only shown protective effects in both in vitro and in vivo models of ALS, but also in clinical trials with ALS patients [8]. Particularly, arimoclomol exerts its neuroprotective effects in ALS by prolonging the activation of heat shock transcription factor-1 [36], a master and early regulator of the HSR [30], thereby increasing the levels of HSPs in motor neurons. In addition, heat shock factor-1 over-expression in ALS mice enhanced proteostasis, as shown by the increased solubility of mutant SOD1 in motor neurons and astrocytes, thus confirming that strategies that activate heat shock factor-1 are valid therapies for ALS [37]. These data certainly make the heat shock factor 1 an appealing potential target to be carefully investigated after the histaminergic challenge in ALS.”

Enhanced Hsp expression is known to affect protein aggregation leading to clearance of aggregates via the proteasome/ubiquitin and/or the autophagy system [38]. In cells under stress conditions, GRP78 activates the final steps of macroautophagy, by binding to misfolded proteins and to SQSTM1/p62, and favoring cargo delivery into the autophagosome with subsequent protein clearance and degradation into amino acids [32]. Moreover, in chaperone-mediated autophagy, Hsp70 with the aid of SQSTM1/p62 selects and carries the ubiquitinated proteins to the receptor complexes on the lysosome to be degraded [6]. Importantly, several SQSTM1/p62 mutations have been identified in patients and contribute to ALS pathology [29]. Our work thus describes that deleteriously increased levels of SQSTM1/p62, which are found in SOD1-G93A cellular models [23,39], can be significantly inhibited by histamine in both motor neurons and microglia, reinforcing the involvement of autophagy proteins in histaminergic protective mechanisms [40].

In ALS, the role of beclin-1 has not been thoroughly investigated yet [41]. Recent studies have shown that autophagic beclin-1 haploinsufficiency has an impact on the disease, leading to protection in SOD1-G93A mice with a significant delay in the onset of the disease and prolonged life span [11]. The histaminergic-mediated actions on autophagy are further corroborated by the strong decrease of beclin-1 that we observed in SOD1-G93A mice spinal cords after histidine treatment. Although preliminary at this stage, these results would suggest not only that the histaminergic signaling participates to the autophagy machinery in ALS, but also that the histamine beneficial actions would proceed through the HSR-autophagy axis.

Although Hsps such as Hsp70 have been shown to be powerful inhibitors of apoptosis, an increase in Hsp expression per se is not indicative of a cytoprotective effect because the activation of the HSR may be a consequence of stress-inducing cytotoxicity [21]. We have shown here that histamine directly increases the expression of anti-apoptotic Bcl-xL both in vitro and in vivo in SOD1-G93A models. This up-regulation occurs concomitantly with an increase of cell survival and mitochondria integrity in SOD1-G93A motor neurons, and with an anti-inflammatory microglia phenotype promoted by histamine [16], thus suggesting a beneficial HSR induction, likely correlated with the neuroprotective role of histamine.

A therapy inducing Hsps is already known to exert neuroprotection for instance in transgenic AD mice by reducing tau pathology and amyloid plaque formation, and also by increasing dendritic spine density [42,43]. As the with histidine entirely prevents dendritic spine loss in SOD1-G93 motor neurons, our results reinforce the relevance of histidine-induced Hsp70 and GRP78 stimulation for preserving motor function. Thus, while confirming that HSR stimulation is beneficial in ALS by prolonging the survival and facilitating the clearance of protein aggregates [6], our observation provides the first evidence that histaminergic signaling maintains intact also the synapse density in the motor cortex, not merely reducing the degeneration of motor neurons.

**Figure 5:**
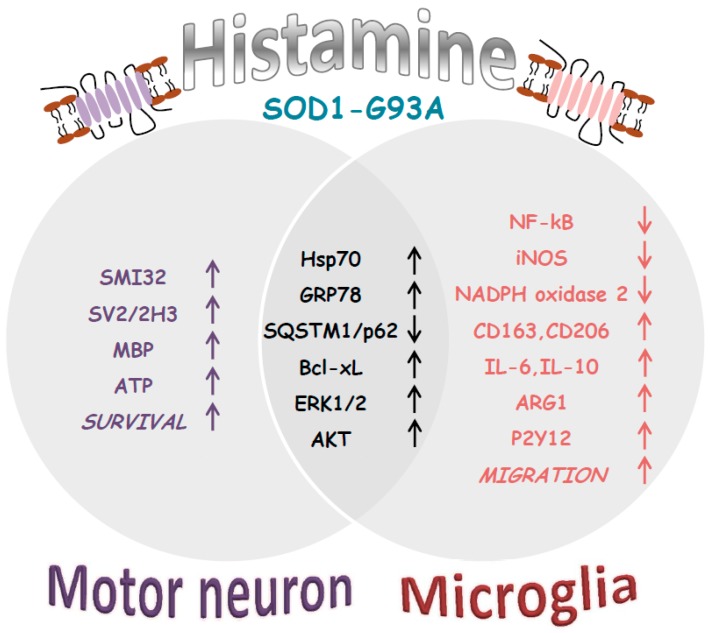
Synopsis of histamine effects on ALS disease features. In SOD1-G93A motor neurons, histamine increases mitochondrial functionality (ATP content) and survival (SMI32), also protecting axons (SV2/2H3; MBP) [16]. In SOD1-G93A microglia, histamine decreases pro-inflammatory markers (NF-kB, iNOS and NADPH oxidase 2), and increases anti-inflammatory markers (CD163, CD206, IL-6, IL-10 ARG1, P2Y12) together with cell migration [17]. In both motor neurons and microglia histamine activates AKT/ERK1/2 [16,17] in addition to Hsps (GRP78, Hsp70) and Bcl-xL responses, while decreasing SQSTM1/p62 (Figure 1, Figure 2, Figure 3 and Figure 4).

A recent work has introduced the novel concept that histaminergic modulation might play a major role in ALS [44]. In particular, histamine-related genes are dysregulated in the cortex and spinal cord from sporadic ALS patients, moreover in SOD1-G93A mice as a function of disease progression, and finally in ALS microglia and motor neuron cultures. An integrative multi-omics approach combining gene expression profiles, copy number variants, and single nucleotide polymorphisms of ALS patients, has further described histamine pathway associations in ALS [16]. Here, we have gathered further information on how the histaminergic signaling is involved with and might work against ALS. In detail, with our work we propose that histamine, by acting on the heat shock response, could simultaneously affect anti-apoptotic and autophagic proteins, thus prompting the microglia toward an M2-like phenotype, and the motor neurons toward survival (Figure 5). These pleiotropic beneficial actions would surely make histamine a good candidate to be further investigated for slowing the progression of ALS and offering a renewed translational power.

## 4. Materials and Methods

### 4.1. Reagents and Antibodies

Histamine dihydrochloride and all other reagents, unless otherwise stated, were obtained from Sigma Aldrich (Milan, Italy). Bcl-xL rabbit antibody (1:100), Beclin-1 mouse monoclonal (1:100), GRP78 (1:100) and HSP70 (1:100) goat antibodies were from Santa Cruz Biotechnology, Inc. (Dallas, TX, USA); SQSTM1/p62 mouse monoclonal antibody (1:500) was obtained from Abcam (Cambridge, UK). GAPDH (1:2500) was from Calbiochem (San Diego, CA, USA). HRP-linked antibodies were from Jackson Immunoresearch (West Grove, PA, USA).

### 4.2. Mice

Adult B6.Cg-Tg(SOD1-G93A)1Gur/J mice expressing high copy number of mutant human SOD1 gene with a Gly93Ala substitution (SOD1-G93A) were obtained from the Jackson Laboratories (Bar Harbor, ME, USA) and maintained in our indoor animal facility. Transgenic hemizygous SOD1-G93A males were crossbred with C57BL/6 females, both maintained on C57BL/6 genetic background. Transgenic progeny was genotyped analyzing tissue extracts from tail tips, as previously described [16]. Animals were housed at constant temperature (22 ± 1 °C) and relative humidity (50%), with a regular 12 h light cycle (light 7 a.m.–7 p.m.). All animal procedures were performed according to European Guidelines for the use of animals in research (86/609/CEE) and the requirements of Italian laws (D.L. 26/2014). The ethical procedure has been approved by the Animal Welfare Office, Department of Public Health and Veterinary, Nutrition and Food Safety, General Management of Animal Care and Veterinary Drugs of the Italian Ministry of Health (protocol number 319/2015PR, approval date 12 May 2015 by the General Management of Animal Care and Veterinary Drugs). All efforts were made to minimize animal suffering and to use the minimum number of animals necessary to obtain reliable results.

### 4.3. Primary Microglia Cultures

Primary microglia cultures from mouse cortex were prepared as previously described [45]. Briefly, P0-P2 SOD1-G93A and wild-type (WT) mice were sacrificed and, after removing the meninges, cortices were minced and digested with 0.01% trypsin and 10 μg/mL DNaseI. After dissociation and passage through 70 μm filters, cells were suspended and plated in DMEM/F-12 media with GlutaMAX™ (Gibco, Invitrogen, Paisley, UK), supplemented with 10% fetal bovine serum (FBS), 100 Units/mL gentamicine and 100 µg/mL streptomycin/penicillin at a density of 62,500 cells/cm^2^. After approximately 15 days, a mild trypsinization in DMEM/F-12 without FBS was performed for 40 min at 37 °C to remove non-microglial cells. The resultant adherent microglial cells (about 98% pure) were washed twice with DMEM/F-12 and kept in 50% mixed glial cells conditioned medium at 37 °C in a 5% CO_2_ and 95% air atmosphere for 48 h until use.

### 4.4. Differentiated NSC-34 Motor Neurons

NSC-34 motor neurons are a mouse neural hybrid cell line produced by fusion of motor neuron-enriched embryonic mouse spinal-cord neurons with mouse neuroblastoma cells. These immortalized motor neuron-like cells resemble motor neurons in many ways, for example, in their generation of action potentials, expression of neurofilament proteins, and acetylcholine synthesis, storage and release [46], and have been widely used as a cellular model of ALS.

NSC-34 cells, stably transfected with WT or mutant human SOD1-G93A DNA [22], were maintained in DMEM/F12 medium supplemented with 10% FBS (Invitrogen) and 1% penicillin/streptomycin. For inducing a differentiated motor neuron-like phenotype, NSC-34 cells were plated in differentiation medium, containing DMEM/F12 supplemented with 0.5% FBS, 1% penicillin/streptomycin and 1% modified Eagle’s medium non-essential amino acids.

### 4.5. Spinal Cord and Cortical Tissue Analysis

Spinal cord and cortex from female SOD1-G93A mice intraperitoneally injected every 48 h with the brain permeable histamine precursor histidine (100 mg/Kg) from disease onset (110 days) until 150 days of age [16] were analyzed for protein expression by western blotting and confocal analysis.

### 4.6. Protein Extraction, SDS-PAGE and Western Blotting

Cells in serum-free medium were harvested in ice-cold RIPA buffer (PBS, 1% Nonidet P-40, 0.5% sodium deoxycholate, 0.1% SDS) added with protease inhibitor cocktail (Sigma Aldrich). Lysates were kept for 30 min in ice and then centrifuged for 10 min at 14,000× *g* at 4 °C. Protein lysates were also obtained by mice lumbar spinal cords segments and cortex in homogenization buffer (20 mM HEPES, pH 7.4, 100 mM NaCl, 1% Triton X-100, 10 mM EDTA) added with protease inhibitor cocktail (Sigma Aldrich). After sonication, lysates were kept for 30 min in ice and then centrifuged for 20 min at 14000× *g* at 4 °C. Supernatants were collected and assayed for protein content by the Bradford detection kit (Bio-Rad Laboratories, Hercules, CA, USA.). Analysis of protein components was performed by Mini-PROTEAN^®^ TGX™ Gels (BioRad) and transferred onto nitrocellulose membranes (Amersham Biosciences, Buckinghamshire, UK). After saturation with blocking agent, blots were probed overnight at 4 °C with the specified antibody, and then incubated for 1 h with HRP-conjugated secondary antibodies and visualized using ECL Advance Western blot detection kit (Amersham Biosciences). Signal intensity quantification was performed by Kodak Image Station analysis software. Values were normalized with mouse anti-GAPDH (1:2500, Calbiochem).

### 4.7. Immunofluorescence and Confocal Microscopy

Cells were fixed for 10 min in 4% paraformaldehyde, permeabilized for 5 min in PBS containing 0.1% Triton X-100. The cells were then incubated for 2.5 h at 37 °C with anti-Bcl-xL (1:100) in 1% BSA in PBS. The cultures were stained for 2 h with Cy2-conjugated donkey anti-rabbit IgG (1:200, Jackson Immunoresearch). Nuclei were stained for 5 min with 1 μg/mL Höechst 33,258 and the cells were mounted and cover-slipped with gel/mount antifading (Biomeda, Foster City, CA, USA).

Spinal cord sections were incubated with the specified antibody in PBS 0.3%Triton X-100 and 2% normal donkey serum at 4 °C for 48 h, washed thoroughly and incubated with appropriate fluorescent-conjugated secondary antibodies for 3 h at room temperature. The secondary antibodies used were Cy5-conjugated donkey anti-goat and anti-rabbit immunoglobulin G (1:100, Jackson Immunoresearch), Cy3-conjugated donkey anti-mouse immunoglobulin G (1:100, Alexa Molecular Probes Inc., Invitrogen). Immunofluorescence was analyzed by means of a confocal laser-scanning microscope (Zeiss, LSM700, Oberkochen, Germany) equipped with four laser lines: 405 nm, 488 nm, 561 nm and 639 nm. The brightness and contrast of the digital images were adjusted using the Zen software 3.0 blue edition (Zeiss).

### 4.8. Golgi Staining of Cortical Motor Neurons and Measurement of Dendritic Spine Density

WT and SOD1-G93A injected with saline or histidine as described above were perfused with 0.9% saline solution; brains were collected and impregnated by immersion in a standard Golgi–Cox solution (1% potassium dichromate, 1% mercuric chloride, 0.8% potassium chromate). After 6 days, the brains were transferred to a 30% sucrose solution for 2 days, and then cut in 100 μm coronal sections. Sections were mounted on gelatinized slides, stained according to the Gibb and Kolb method [47] and coverslipped. Fully impregnated pyramidal neurons lying in layer V of M1 were selected and analyzed using the software Neurolucida (Microbrightfield, Williston, VT, USA) connected to an optical microscope DMLB Leika. From each brain, 5–6 fully impregnated pyramidal neurons lying in layer V of M1 and displaying dendritic tree without truncations were selected, under 20× magnification. On each neuron, five 30–100 μm dendritic segments of secondary and tertiary branch orders were randomly selected for spine counts and analyzed under 100× magnification. A computer-based neuron tracing system (Neurolucida, Microbrightfield) connected to an optical microscope DMLB Leika was used to trace dendritic segments and spines. All protrusions, with or without bulbous expansion, but no longer than 2 μm, were counted as spines, if they were continuous with the dendritic shaft. The spine density (number of spines/1 μm segment) was averaged for a neuron mean.

### 4.9. Data Analysis

Data are presented as mean ± standard error of the mean (S.E.M.). Statistical differences were verified by one-way ANOVA using MedCalc (Medcalc Software, Mariakerke, Belgium) followed by individual post-hoc comparisons (Fisher’s PLSD). *p* < 0.05 was considered significant.

## Abbreviations

ALS	Amyotrophic lateral sclerosis
CNS	Central nervous system
SOD1	Superoxide dismutase 1
HSR	Heat shock response
Hsps	Heat shock proteins
NFκB	Nuclear factor kappa-light-chain-enhancer of activated B cells
NO	Nitric oxide
WT	Wild-type
